# Preparedness against pandemic influenza: Production of an oil-in-water emulsion adjuvant in Brazil

**DOI:** 10.1371/journal.pone.0233632

**Published:** 2020-06-03

**Authors:** Milena Apetito Akamatsu, Vitor Anselmo Sakihara, Bianca Pereira Carvalho, Aline de Paiva Abrantes, Maria A. Sakauchi Takano, Eduardo Alfredo Adami, Fernando Seiji Yonehara, Patrícia dos Santos Carneiro, Stefanni Rico, Alessandra Schanoski, Maurício Meros, Adrian Simpson, Tony Phan, Christopher B. Fox, Paulo Lee Ho

**Affiliations:** 1 Divisão BioIndustrial, Serviço de Bacteriologia, Instituto Butantan, São Paulo, Brazil; 2 Divisão BioIndustrial, Laboratório de Influenza, Instituto Butantan, São Paulo, Brazil; 3 Controle de Qualidade, Instituto Butantan, São Paulo, Brazil; 4 Laboratório de Bacteriologia, Instituto Butantan, São Paulo, Brazil; 5 Divisão BioIndustrial, Instituto Butantan, São Paulo, Brazil; 6 Infectious Disease Research Institute, Seattle, Washington, United States of America; University of South Dakota, UNITED STATES

## Abstract

Increasing pandemic influenza vaccine manufacturing capacity is considered strategic by WHO. Adjuvant use is key in this strategy in order to spare the vaccine doses and by increasing immune protection. We describe here the production and stability studies of a squalene based oil-in-water emulsion, adjuvant IB160, and the immune response of the H7N9 vaccine combined with IB160. To qualify the production of IB160 we produced 10 consistency lots of IB160 and the average results were: pH 6.4±0.05; squalene 48.8±.0.03 mg/ml; osmolality 47.6±6.9 mmol/kg; Z-average 157±2 nm, with polydispersity index (PDI) of 0.085±0.024 and endotoxin levels <0.5 EU/mL. The emulsion particle size was stable for at least six months at 25°C and 24 months at 4–8°C. Two doses of H7N9 vaccine formulated at 7.5 μg/dose or 15 μg/dose with adjuvant IB160 showed a significant increase of hemagglutination inhibition (HAI) titers in sera of immunized BALB/c mice when compared to control sera from animals immunized with the H7N9 antigens without adjuvant. Thus the antigen-sparing capacity of IB160 can potentially increase the production of the H7N9 pandemic vaccine and represents an important achievement for preparedness against pandemic influenza and a successful North (IDRI) to South (Butantan Institute) technology transfer for the production of the adjuvant emulsion IB160.

## Introduction

It is recognized that preparedness for public health emergencies, such as pandemics, earthquakes or terrorist attacks, should include the development and setup of appropriate countermeasures ready for rapid activation [1–5]. Influenza pandemics are unpredictable but recurring events can have severe consequences on human health and on societies worldwide. Advanced planning and preparedness are critical to help mitigate the impact of a global pandemic [6].

Global influenza vaccine manufacturing capacity has been enhanced from 2.6 billion doses in 2009 to 5.1 billion doses in 2016 by creating new production sites or increased production scale, representing a significant improvement [7]. Antigen sparing by employing adjuvants also represents a key technology for global pandemic influenza preparedness [8]. Moreover, during a pandemic, adjuvants are particularly beneficial for influenza vaccines when a rapid and a higher immune response is required or there is a need to improve the overall immune response, especially in patients with impaired immunological responses such as the pediatric and elderly populations [9, 10].

Oil-in-water emulsions have been shown to effectively and safely induce immune responses to influenza antigens, enabling antigen sparing and cross-clade neutralizing antibody responses [11–15]. Several clinical trials have compared the safety and immunogenicity of emulsion-adjuvanted vaccines with those of conventional split influenza vaccines in elderly subjects [16–19]. The first adjuvant to be included in a licensed seasonal influenza vaccine was MF59^®^ (Fluad was licensed in Europe in 1997 for older adults). More recently, MF59^®^-containing vaccines have been approved for other age groups including children (6–24 months in Canada) and for pandemic influenza vaccines [10]. MF59^®^ is an oil-in-water emulsion composed of squalene and the non-ionic surfactants polysorbate 80 and sorbitan trioleate. MF59^®^ enhances the humoral and cellular immune response in humans, not only to influenza but also to vaccination with HSV, HIV and other antigens, showing an overall excellent safety profile with millions of doses administered among all age groups [16–21]. It is important to note that no association between narcolepsy and MF59^®^-containing vaccines has been described [22].

The World Health Organization (WHO) encourages countries to develop and implement national pandemic preparedness plans to mitigate the health and social effects of a potential pandemic [23–24]. Increasing global pandemic influenza vaccine manufacturing capacity is considered strategic by WHO and the Biomedical Advanced Research and Development Authority (BARDA) of the US Department of Human and Health Services (HHS) [8, 24]. However, the limited financial and technical resources in developing countries make the preparedness for a pandemic more challenging in these regions [23]. In this regard, production of appropriate adjuvant for pandemic influenza is key in order to spare vaccine antigen doses as well as to increase protective immune responses.

**Fig 1:**
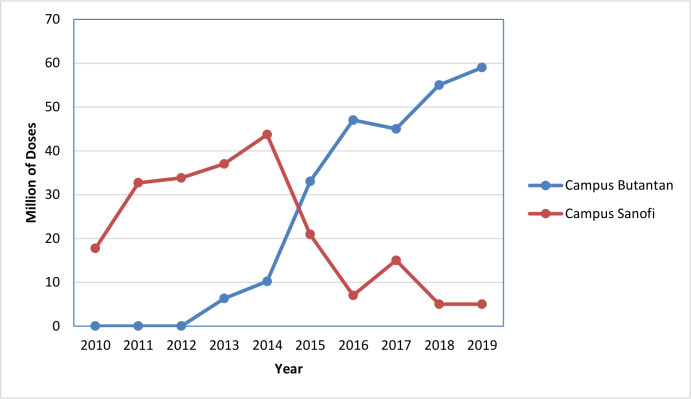
Production of seasonal trivalent influenza vaccine by Butantan Institute. Part of the production was supplied by Sanofi-Pasteur (campus Sanofi-Pasteur) and part was produced at Butantan Institute (campus Butantan) over the years.

The production of influenza vaccine in Brazil is carried out by Butantan Institute, a public São Paulo State Institution. The industrial infrastructure is able to produce both seasonal and pandemic influenza vaccines, such as H1N1 and H5N1 [25–26]. Butantan Institute received the certificate of current good manufacturing practices (cGMP) from the Brazilian Regulatory Agency (ANVISA) and delivered the first lot of egg-based seasonal trivalent split influenza vaccine entirely produced in Brazil for the 2013 campaign. This was the first successful technological transfer completed in Brazil between Sanofi-Pasteur and the Butantan Institute [27–28]. For the 2019 vaccination campaign, Butantan Institute produced and delivered 59 million doses of trivalent seasonal influenza for the Brazilian Ministry of Health, representing 92% of the total demand (64 million doses), with the remaining doses supplied by Sanofi-Pasteur (Fig 1).

In order to further enhance pandemic preparedness, Butantan Institute sought to establish local adjuvant manufacturing capacity. Under the support of WHO and BARDA, Butantan Institute received general technical training in 2015 from the Infectious Disease Research Institute (IDRI) for the production and characterization of oil-in-water emulsions. Butantan Institute then applied its newly obtained expertise to develop the production of IB160, a squalene based emulsion adjuvant similar if not identical to MF59^®^ adjuvant as a key part of the preparedness approach for pandemic influenza in the Southern hemisphere. The production and stability studies of a squalene based oil-in-water emulsion, adjuvant IB160, and the immune response of the H7N9 vaccine combined with IB160 is described here as a successful North (IDRI) to South (Butantan Institute) hemisphere tech-transfer. To evaluate the adjuvant effect of IB160, mice were immunized with H7N9 antigens combined or not with IB160 and the differences in hemagglutination inhibition (HAI) titers among immunized and control animals were analysed.

## Material and methods

### Ethical statement

The animal studies was performed according to the guidelines outlined by the Brazilian National Council for Control of Animal Experimentation (CONCEA). Experimental protocols were approved by the Ethic Committee on Animal Use of the Butantan Institute (CEUAIB) (protocol numbers CEUAIB 1301/14). The mice were obtained by the animal facility from the Medical School, University of São Paulo. Group of five animals were housed per cage inside a ventilated cabinet under controlled temperature and light cycle (12/12 hours, light/dark cycle) with daily monitoring in a BSL2 animal facility. Food and water were given ad libitum. The euthanasia of the animals was done using a lethal doses of xylazine hydrochloride and ketamine hydrochloride solution (60mg / kg of xylazine and 300mg / kg of ketamine) via intraperitoneal, as approved in the CEUAIB protocol. Monitoring and manipulation was done by trained personnel.

### Production of adjuvant IB160

**Fig 2:**
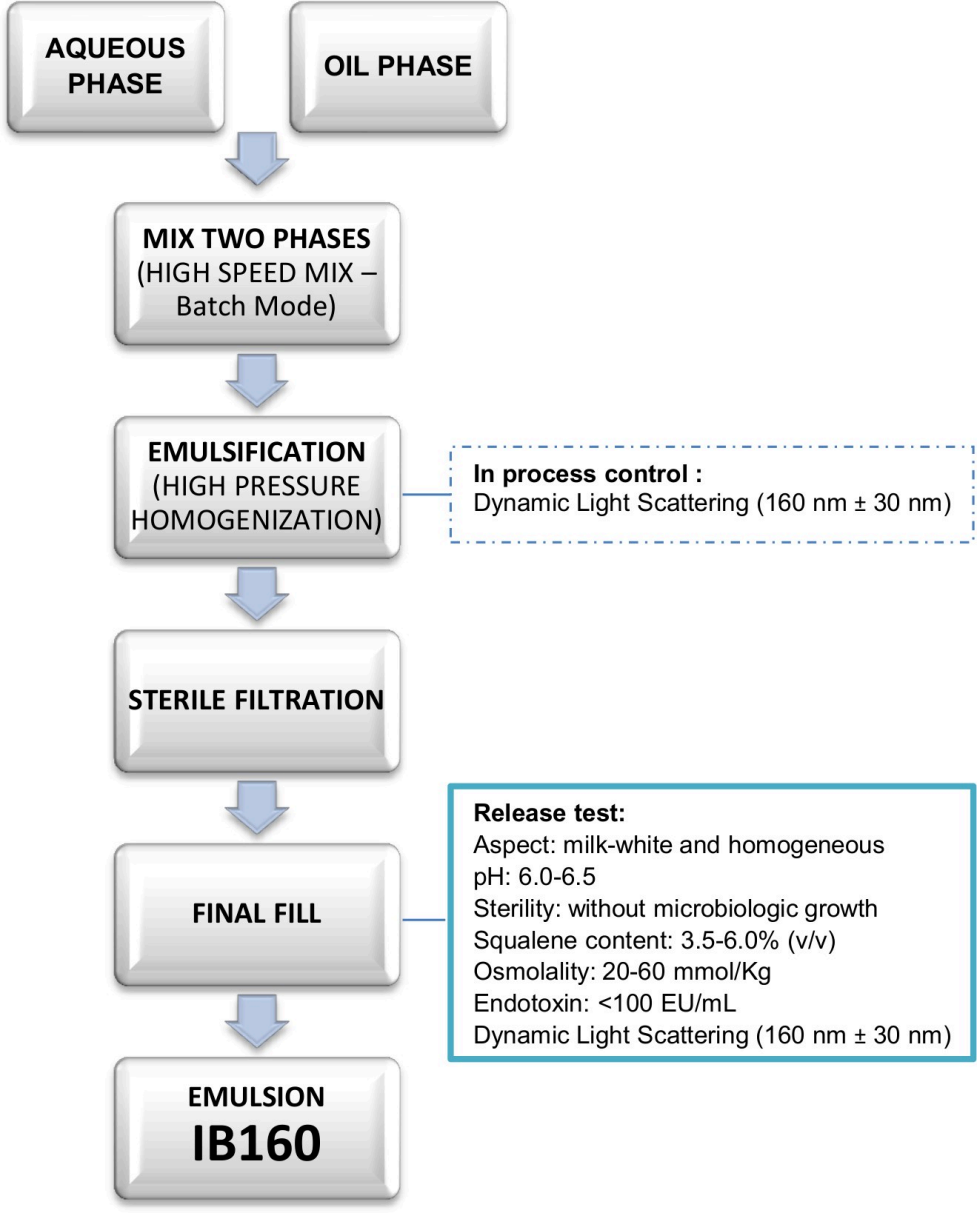
Process flow diagram of emulsion IB160 production.

The oil in water emulsion IB160 was prepared as presented in Fig 2 and basically according to Ott, 2000 [29]. It consists of an aqueous phase (10 mM citrate buffer (5.29 mg/ml sodium citrate dihydrate and 0.34 mg/ml citric acid monohydrate) with pH between 6.0 and 6.5, 5.3 mg/ml polysorbate 80 (Tween® 80), and an oil phase (43 mg/ml squalene oil and 4.78 mg/ml sorbitan trioleate (Span^®^ 85)). The aqueous and oil phases were mixed together in a high speed mixer batch mode (L5M-A Laboratory Mixer, Silverson Machines) at 10,000 rpm for 25 min. The emulsification process was performed in a high pressure homogenizer (M-110EH-30K Processor, Microfluidics), operating at ~15,000 psi, processed repeatedly 10 times, until the desired particle size was obtained. As an in-process control, particle size was measured by dynamic light scattering (Zetasizer Nano-S, Malvern Instruments) [30]. The emulsion was filter-sterilized through a 0.22 μm PVDF filter using a peristaltic pump. Following filtration, the emulsion was filled at 1.5-mL volume in 7.5-mL glass vials, which were closed with polytetrafluoroethylene (PTFE)-coated chlorobutyl stoppers and aluminum caps.

For characterization of the filled IB160, we performed several quality control tests: physical appearance, pH, sterility testing, osmolality, endotoxin, squalene content, and particle size. The physical appearance is a visual inspection performed manually under adequate lighting by gently swirling a emulsion sample in the glass vial and assessing whether the appearance is milky-white and homogeneous. For sterility testing, the samples were cultivated in thioglycolate and TSB medium incubated at 32.5°C ± 2.5°C and 22.5°C ± 2.5°C, respectively, for 4 days according to WHO [31]. Squalene content was characterized by RP-HPLC (Agilent 1200 HPLC, Agilent Technologies) with charged aerosol detection (Corona CAD, Thermo Fisher) and an Atlantis T3 C18 column (4.6 X 250 mm, 5 μm, Waters, Milford, MA) at 30°C. The mobile phase consisted of solution A (75:15:10 of Methanol:Chloroform:Water composed of 1% acetic acid; respectively) and solution B (1:1:0.02 of Methanol:Chloroform:Acetic acid) at a flow rate of 1.0 mL/min in a 30 min gradient consisting of mobile phases A and B. Squalene concentration measurements were made by interpolation from a standard curve integrating the area of the peaks. Standard concentrations of squalene were determined gravimetrically.

Osmolality was assessed by vapor pressure osmometry (Vapro 5600, Wescor) with the instrument calibrated with vendor-supplied standards prior to measurement of triplicate samples, which were pipetted to the sample holder as undiluted 10-μl aliquots as previously described [30]. The endotoxic activity was determined by the chromogenic kinetic method (Charles River Laboratories) and the results expressed in EU/mL. Particle size was measured by dynamic light scattering after 1:100 dilution in water (Zetasizer Nano S, Malvern Instruments), reported as Z-avg.

### The stability of IB160

The filled emulsion vials were stored at 5.0°C ± 3.0°C for long-term stability studies (24 months) and 25.0°C ± 2.0°C with 60% ± 5% relative humidity for accelerated stability studies (6 months). The vials were placed in normal and inverted positions. The samples were analyzed at time 0, 3 and 6 months for the accelerated stability study and time 0, 3, 6, 9, 12, and 18 months for the long-term real-time stability study.

### Influenza H7N9 production and IB160 adjuvanted H7N9 vaccine formulation

The proof of concept study of the adjuvant bioactivity was performed using pre-pandemic influenza H7N9 vaccine strain. Influenza H7N9 vaccines were produced at Butantan’s industrial plant, using the A/Shanghai/2/2013(H7N9)-PR8-IDCDC-RG32A.3, supplied by the United States Centers for Disease Control and Prevention (CDC) through the Global Influenza Surveillance and Response System (GISRS). For the production of the split and inactivated vaccines, the working seed virus was grown in 10–11 days embryonated hen’s eggs (144,000 embryonated eggs, purchased from Globoaves, BR) and incubated at 33–35°C (Incubator CASP model Ug 124HT, BR, capacity for 123,480 eggs) for 60 hours. After incubation, the eggs were chilled overnight at 2–8°C and the allantoic liquid (AL) was harvested after removal of the top of the shell with a knife cut using a proprietary designed equipment by Butantan Institute and assembled by FAE (http://www.faesystem.com.br/novo/), clarified by continuous centrifugation and concentrated by diafiltration (cut off 300 kDa). The concentrated virions were purified by two consecutive industrial ultracentrifugation steps of sucrose gradient, collected, diluted with phosphate buffer and split by Triton X-100 (0.5% final concentration). The split H7N9 was further clarified by continuous centrifugation and diafiltration (cut off 50 kDa, using phosphate buffer and 10 diafiltration volumes). After appropriate dilution, formaldehyde was added to 0.01% final concentration for inactivation. The H7N9 antigen was filtered in 0.22 μm PVDF filter and considered concentrated H7N9 vaccine antigen bulk. Quality control of H7N9 vaccine antigen was performed (virus inactivation, sterility, endotoxin content, pH, nitrogen content, residual Triton X-100, residual formaldehyde, protein content, visual aspect, hemagglutinin identity, ovalbumin content, hemagglutinin content by single radial immunodiffusion assay) according to the WHO guidelines [32]. Prior to immunization, the emulsion IB160 was mixed with the H7N9 antigen preparation in 1:1 v/v and immediately inoculated in the animals. The H7N9 vaccine formulation was prepared at 3 different final concentrations by first diluting with phosphate buffer saline prior to mixing with IB160: 3.75 μg/dose, 7.5 μg/dose and 15 μg/dose, with or without IB160 oil-in-water emulsion adjuvant.

### Immunization and hemagglutination inhibition (HAI) titers

The different vaccine formulations were administered by the subcutaneous route to groups of 5 BALB/c female mice, 4 weeks old, in a total volume of 0.3 mL split between two different sites of inguinal area. Animals were bled from the retro-orbital plexus, under local anesthesia with 5% proxymetacaine chloride eye drops (Alcon, Texas, USA), after 21 days and sera separated for the determination of hemagglutination inhibition (HAI) titer by the inhibition of guinea pig red blood cells (RBCs) agglutination by the sera, as previously described [33], following the recommendations by WHO (https://www.who.int/influenza/gisrs_laboratory/cnic_serological_diagnosis_hai_a_h7n9.pdf). Hemagglutination occurs when the red blood cells (RBC) control has settled completely. This is recorded using a "+" symbol. When a portion of the RBC is partially agglutinated or partially settled, a "+/-" symbol is used. In the absence of hemagglutination, tear-shaped streaming of erythrocytes which flow at the same rate as RBC controls is observed. A second dose was administered at the 21st day after the first dose, and the animals were bled as described above after 21 days of the second dose for the determination of HAI titer in the sera. An HAI titer ≥ 1:40 was considered positive seroconversion [34].

### Statistical analysis

Statistically significant differences between the animal groups immunized with vaccines with or without adjuvant at the same dose were determined by analysis of variance (Two-way ANOVA) with Bonferroni’s multiple tests, and differences between the sera from the experimental group of animals with their preimmune sera were determined by analysis of variance (Two-way ANOVA) with Bonferroni’s correction, using GraphPad Prism version 6.05 for Windows (GraphPad Software, La Jolla, CA, USA; www.graphpad.com). All tests were considered significant when p ≤ 0.05.

## Results

**Fig 3:**
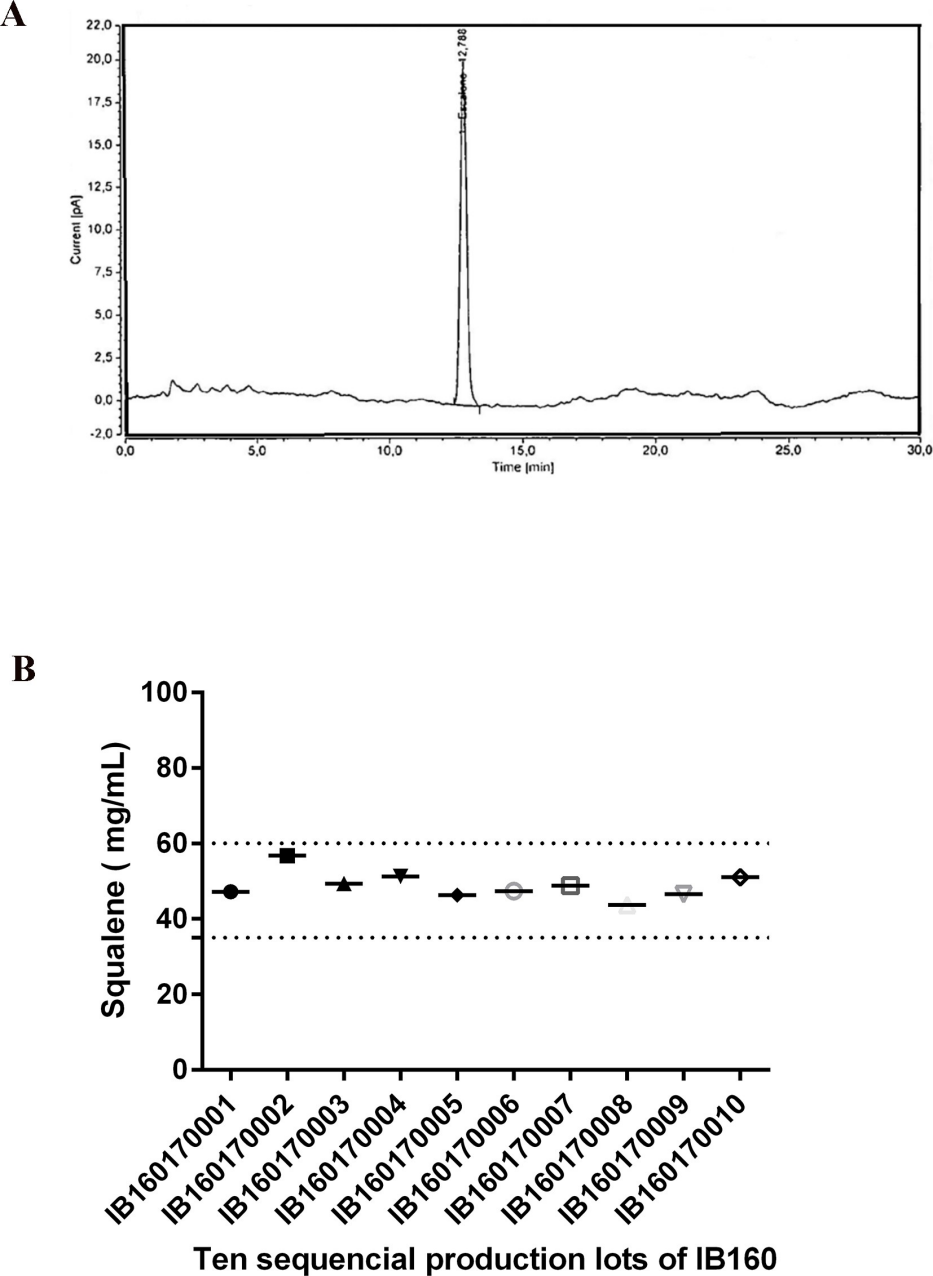
Determination of squalene content by RP-HPLC using a C18 column from the 10 consecutive production lots. The analysis shown (see Materials and Methods) is representative of all the batches of IB160 produced (A). The squalene content from all the lots are shown in (B).

**Fig 4:**
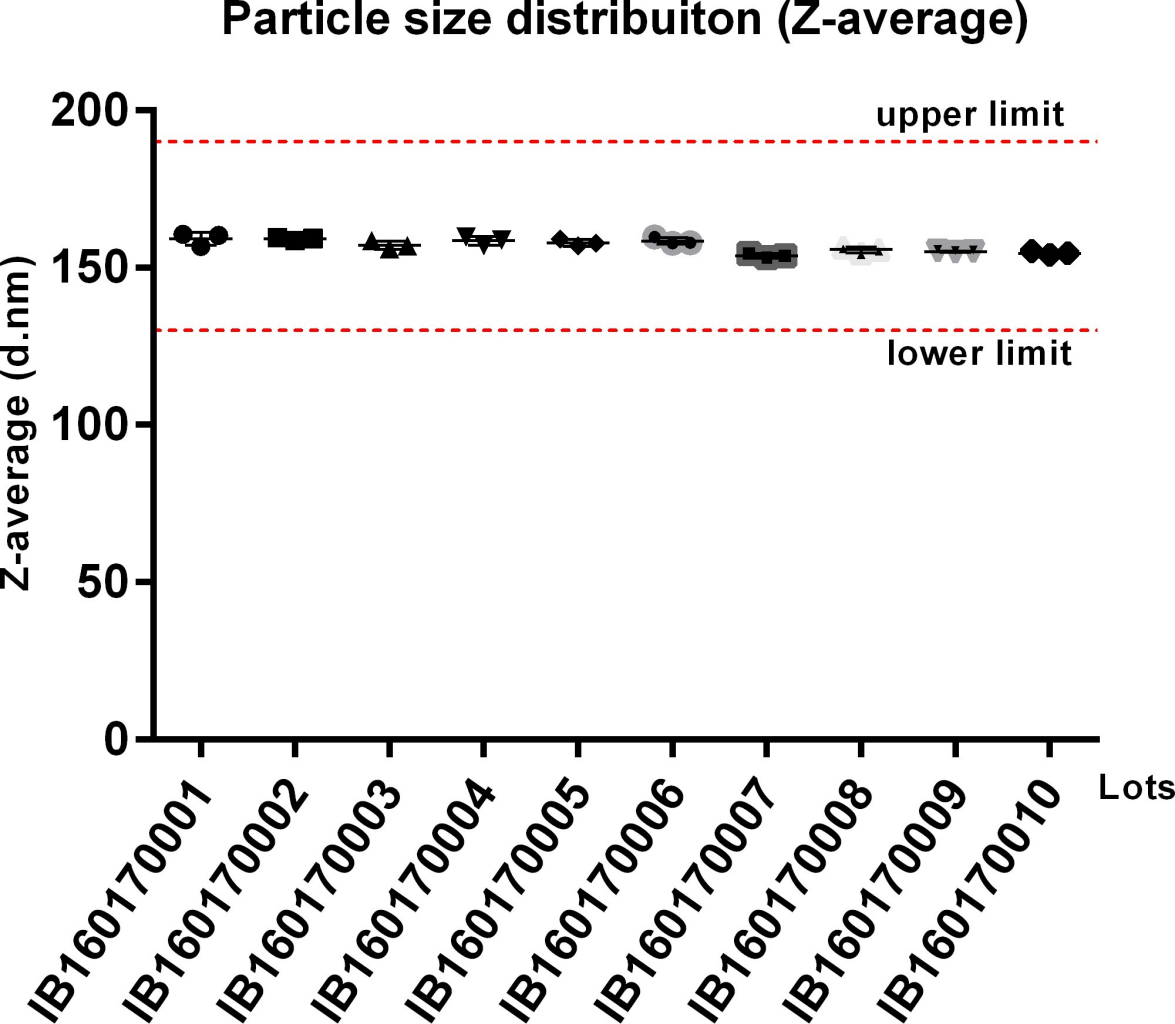
Average particle size of IB160 from 10 consecutive production lots. The measurements were performed from 3 different vials. The dotted red lines indicate the established acceptable particle size range.

Ten distinct batches of IB160 were produced at a volume of 2 L each. This volume allows the theoretical production of 8,000 adjuvanted vaccine doses/batch assuming a 1:1 v/v mix with antigen and a 0.5 ml total injection volume (0.25 mL antigen + 0.25 mL IB160). The average results of the 10 lots were pH 6.4±0.05, squalene 48.8±0.03 mg/mL, osmolality 47.6±6.9 mmol/kg, Z-average 157±2 nm with a PDI of 0.085±0.024. The production of the IB160 emulsion was consistent as illustrated by the homogeneity of the squalene content (Fig 3) and of the produced particle sizes (Fig 4).

**Fig 5:**
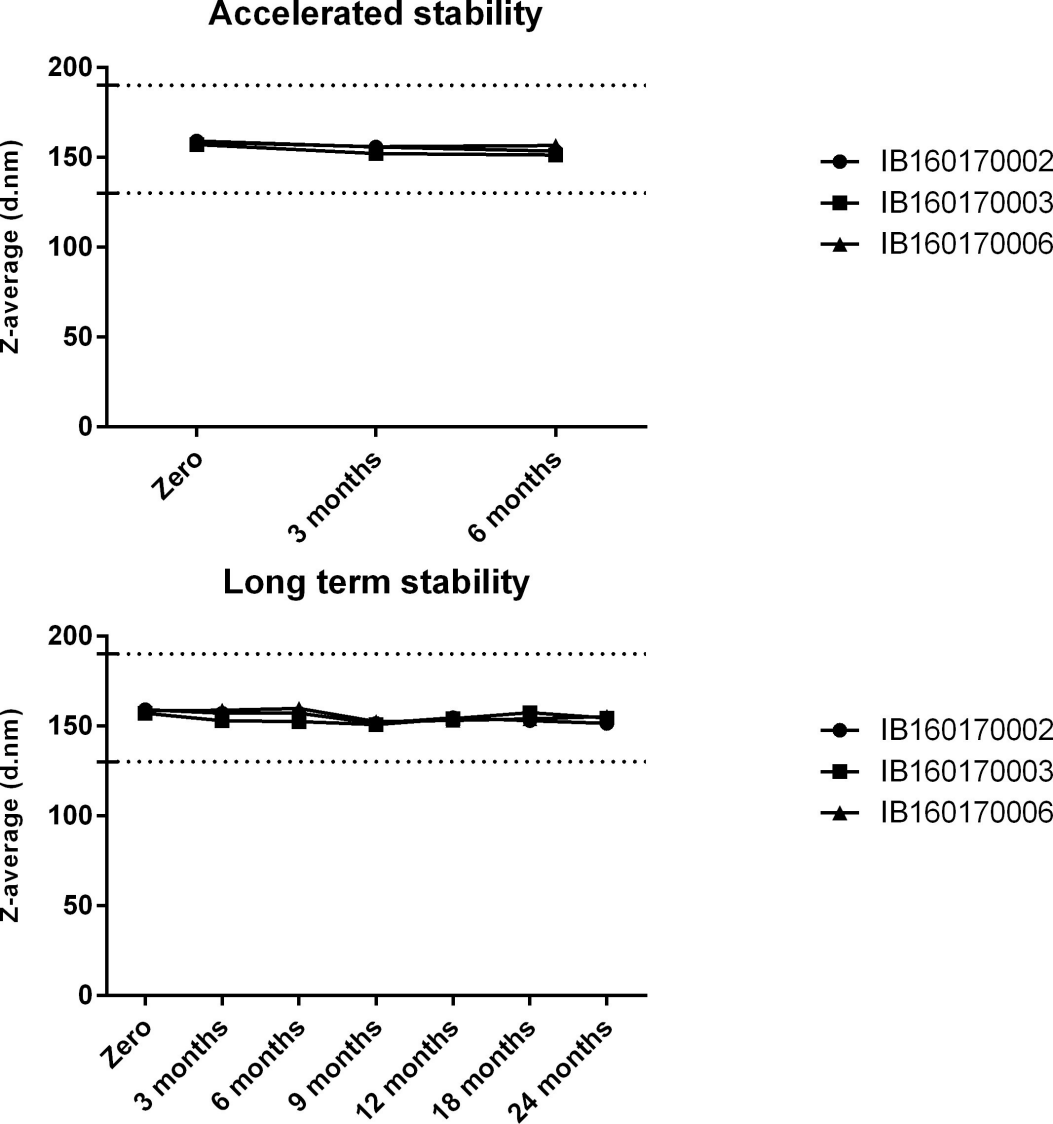
Stability studies of IB160 emulsion. Accelerated (25°C ± 2°C) and long-term (5°C ± 3°C) stability studies were performed with the same 3 lots. The dotted lines indicate the acceptable range of the particle sizes for IB160 emulsion.

**Fig 6:**
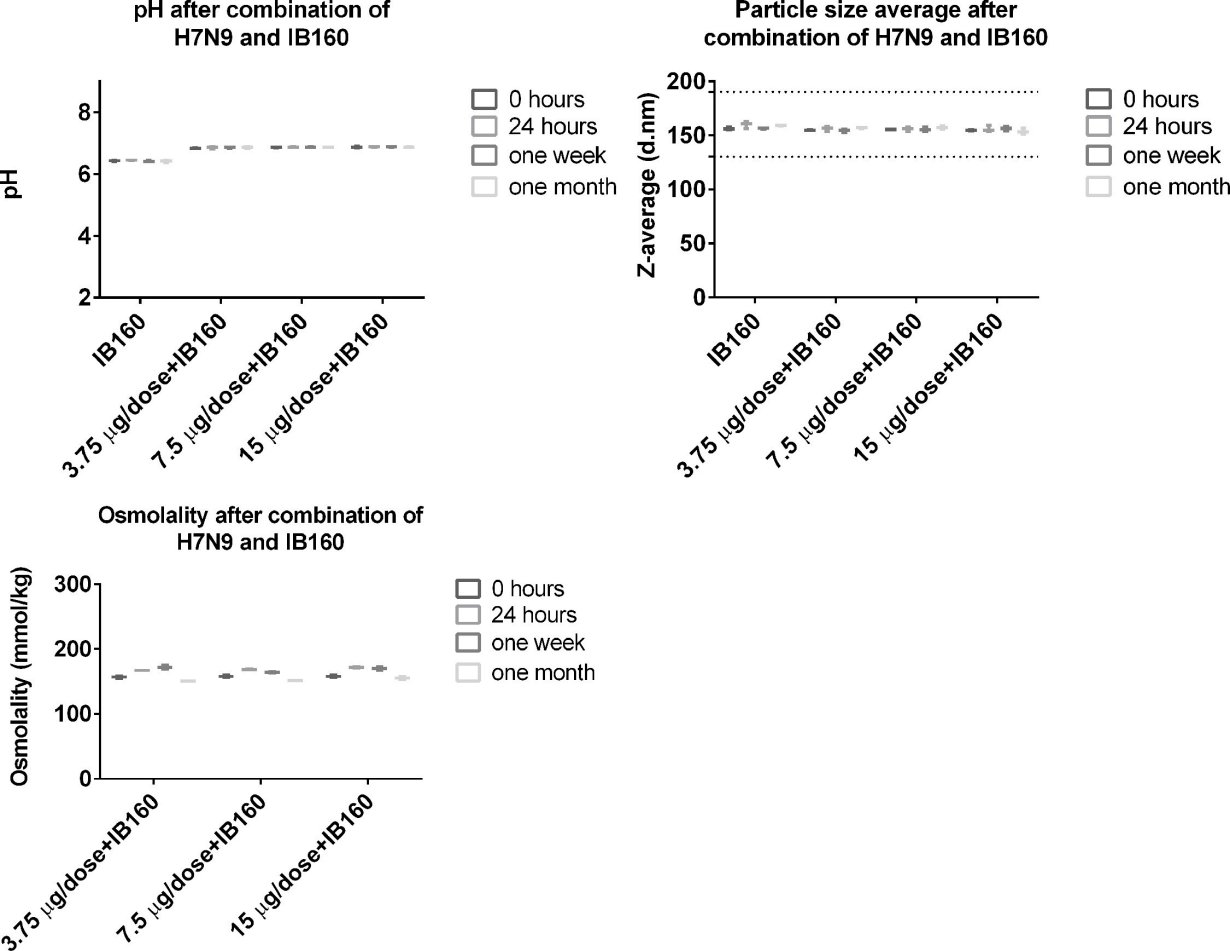
pH, osmolality and particle size average after combination of H7N9 antigen with IB160. The 3 doses of H7N9 (3.75 μg, 7.50 μg and 15.0 μg) were combined with IB160 and the pH, osmolality and particle size were analyzed at 0, 24 hours, one week and one month after mixing.

Stability of the emulsion IB160 was determined by monitoring particle size in 3 different lots. The particle size showed little or no change for at least six months in the accelerated stability studies at 25°C ± 2°C (Fig 5). The same was observed in the long-term stability studies at 5°C ± 3°C, for at least 24 months (Fig 5, study still in progress). Moreover, when IB160 was combined with H7N9 antigen, pH and the particle size were homogenous for at least one month at 5°C ± 3°C. It is also important to note that the initial pH of IB160 (6.5) is increased to 6.85 when combined with the various concentrations of H7N9 antigen (Fig 6). H7N9 antigen when combined with IB160 did not affect the particle size of IB160 (Fig 6).

**Fig 7:**
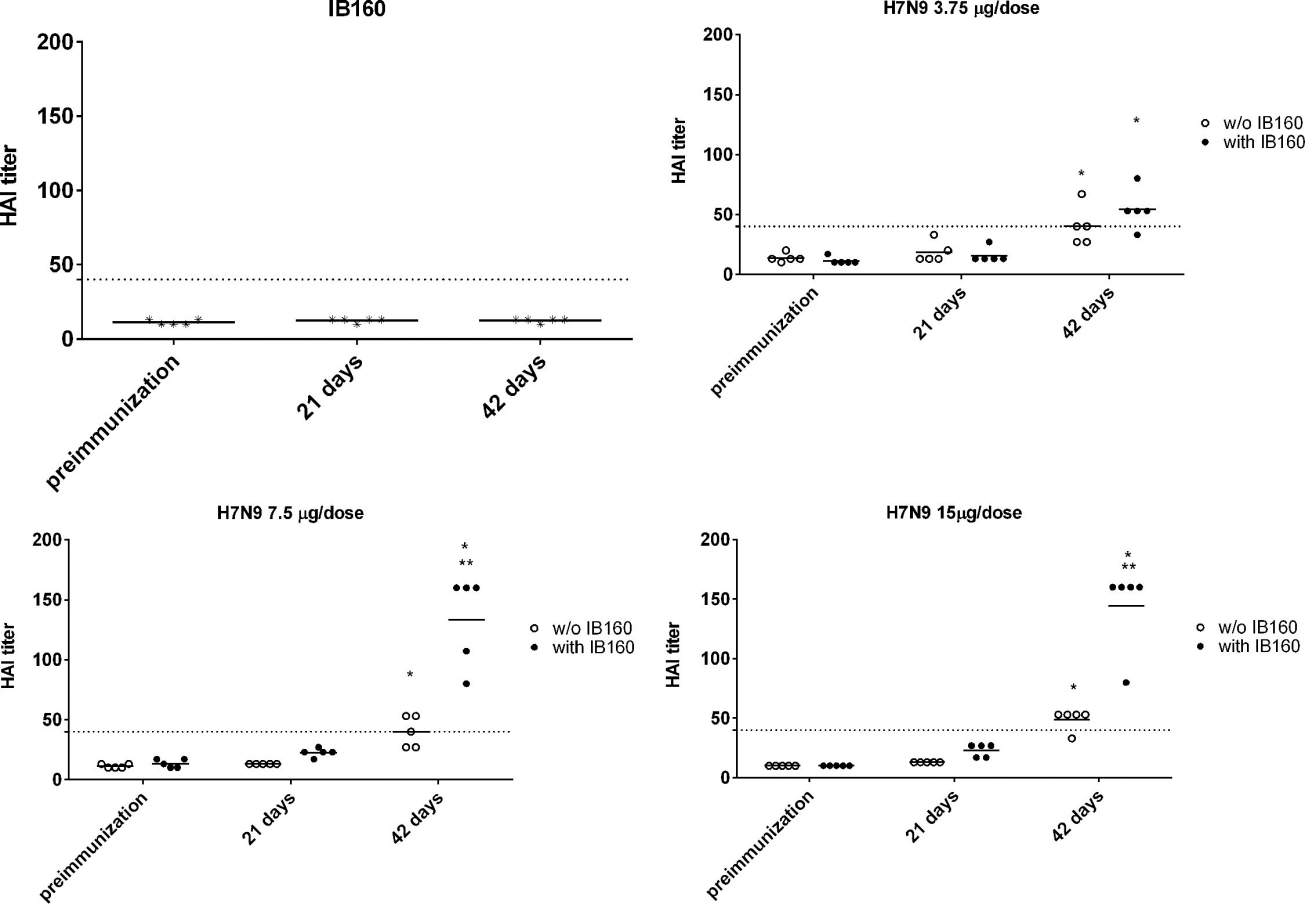
Inhibition of hemagglutination (HAI) by H7N9 antigen. Using sera from immunized mice with H7N9 antigen combined with or without the oil-in-water emulsion adjuvant IB160. Mice immunized twice subcutaneously at day 1 and day 21 with IB160 alone, 3.75 μg, 7.50 μg or 15.0 μg of H7N9 antigen with or without IB160. Sera were collected from the animals at day 1, before the first immunization; day 21, before the second immunization; and day 42, representing 21 days after the second immunization. * Statistical difference in HAI titers when comparing the sera from the experimental group of animals with their preimmune sera; ** Statistical difference in HAI titers when comparing the sera from experimental group of animals immunized with H7N9 antigen combined with IB160 with the sera from experimental group of animals immunized with only the H7N9 antigen at the same dose.

Sera from all immunized mice with two doses of H7N9 vaccine alone or combined with IB160 (Lot IB160170008) were collected after 21 days of the first and second immunizations. As shown in Fig 7, HAI titers were only achieved after the second dose in the group of animals immunized with 7.5 or 15.0 μg of H7N9 with or without IB160 and also in the group of animals immunized with 3.75 μg of H7N9 with IB160. The HAI titers of animals from the 3.75 μg of H7N9 with IB160 after the second dose are comparable to those from animals immunized with 15.0 μg of H7N9 antigen (without IB160) after the second dose. However, statistically higher titers were obtained when 7.5 or 15.0 μg of H7N9 vaccine was combined with the adjuvant IB160 when compared with the control groups at the same doses that did not receive IB160. This result showed that IB160 is an effective oil-in-water emulsion adjuvant in BALB/c mice. During the assay period, no adverse events were observed in all the animals.

## Discussion

In case of an influenza pandemic, manufacturers will shift their production lines from seasonal influenza vaccines to pandemic vaccines, taking advantage of existing production plants and expertise. As soon as the pandemic influenza vaccine strain becomes available, the production will initiate but some time will still be needed for the vaccines to become available for use. In this scenario, the population of the manufacturing countries will be the first to benefit from these first production lots. Excess doses will then be used for the neighbor and non-neighbor countries. Therefore, as soon as the vaccines become available, the negative impact of the pandemic influenza will be decreased mainly according to the speed and access of vaccine supplies, the volume of vaccines used by the population, and the spread of the disease. In this sense, adjuvanted vaccines will be strategic to spare the vaccine doses, thereby increasing the production and availability of vaccines globally, especially for developing countries that, in general, do not maintain local seasonal influenza vaccine production capabilities.

Pandemic influenza vaccines can be very poor immunogens, being necessary the use of adjuvants, high dose of the antigens (45–90 ug of the pandemic HA versus 15 ug of the seasonal HA influenza) and/or multiple vaccinations [35]. For example, H7N9 influenza is considered a potential pandemic threat. Therefore, efforts have been made for H7N9 pandemic influenza preparedness. However, split vaccine preparations of H7N9 were shown to be very poor immunogens. The amount of seasonal influenza antigens used is 15 μg of each strain in a unique application. In contrast, for H7N9 vaccines, one or two doses ranging from 3.75 to 90 μg of antigen were ineffective to induce HAI titers ≥ 1:40 in mice, ferret and humans. A desirable response was only obtained with the use of adjuvants and in a two-dose regimen [36–40]. Thus, preparedness for a pandemic influenza must also involve the capacity to produce a compatible adjuvant in order to provide effective vaccines.

Technology transfer for the production of a stable squalene emulsion (SE) adjuvant was previously implemented at The Cantacuzino Institute in Bucharest (Romania) by the Infectious Disease Research Institute (IDRI, Seattle, USA) and also by the University of Lausanne (Switzerland) to Bio Farma (Indonesia) [30, 41]. These efforts were also part of BARDA/WHO-supported pandemic influenza preparedness which were designed to enable these Institutes to produce enough doses of 15 μg/dose of monovalent vaccine to protect their populations in case of a pandemic influenza. Likewise, we report here the production of a squalene emulsion by a developing country institution. In this case, Butantan Institute first received general training in oil-in-water emulsion know-how that was subsequently applied to develop the production of IB160, an oil-in-water emulsion similar if not identical to MF59. MF59 is already used in seasonal influenza vaccines for elderly (for example, FLUAD®). FLUAD® is prepared by combining the three virus antigens with the MF59C.1 adjuvant. After combining, FLUAD is a sterile, milky-white suspension supplied in 0.5 mL single-dose prefilled syringe and each 0.5 mL dose contains 15 ug of hemagglutinin (HA) from each of the three recommended influenza strains and the MF59 adjuvant (9.75 mg of squalene, 1.175 mg of polysorbate 80, 1.175 mg of sorbitan trioleate, 0.66 mg of sodium citrate dihydrate and 0.04 mg of citric acid monohydrate) at pH 6.9–7.7. In our case, IB160 has the same components but with slightly different concentrations in a 0.5 ml vaccine volume: 10.75 mg squalene, 1.325 mg of polysorbate 80, 1.195 mg of sorbitan trioleate, 1.323 mg sodium citrate dihydrate and 0.0848 mg of citric acid monohydrate) at pH 6.0–6.5. The production of IB160 was robust and resulted in a very consistent and homogenous emulsion, with an average size particle of 157 nm (Fig 4). The average results obtained from 10 consistency lots of pH, squalene content, osmolality, Z-average and PDI of the emulsion can also be used as criterion for product release limits. Furthermore, this emulsion was stable over 24 months at 5°C ± 3°C (Fig 5) and, when combined with H7N9 antigen, pH, osmolality and particle size remained stable (Fig 6). Osmolality values higher than 600 mOsm/kg has been associated to hypertonicity-induced pain. Our results showed a value lower than this limit and the pH were also at physiological range, desirable parameters in the vaccine design to avoid pain in the local injection site [42].

Furthermore, IB160 also presented the expected adjuvant effect when combined with split H7N9 influenza vaccine (Fig 7). The immune response measured by HAI is comparable to other reports in the literature [36–40]. Although a single IB160 adjuvanted H7N9 dose was ineffective to induce a HAI titer ≥ 1:40 in any of the conditions tested, a two dose regimen of 7.5 μg/dose was able to elicite HAI titer ≥ 1:40 (Fig 7). If the 7.5 μg dose can be demonstrated through future studies to be directly translated to humans, it means that the use of IB160 would make possible the production of 350 million doses of H7N9 monovalent vaccines at Butantan Institute (assuming the same production yield of the trivalent seasonal influenza vaccine), which almost covers two doses for the entire population of Brazil (total population ~200 million). Moreover, with 45% increased seasonal production capacity planned for the 2020 campaign, Butantan Institute would then have sufficient capacity to cover two doses of an adjuvanted pandemic vaccine at a 7.5-μg dose for the entire population of Brazil.

The successful development of IB160 production by Butantan Institute is strategic for the preparedness of pandemic influenza to complement the capacity of the H7N9 antigen production in alignment with WHO strategies [24]. The challenge in the next years is to establish the scale up and commercial production conditions for IB160 to achieve self-sufficiency in case of a pandemic influenza in Brazil as well as a potential safeguard for neighboring countries in Latin America. To this end, a Phase 1 clinical trial evaluating the safety and immunogenicity of split inactivated H7N9 vaccine with IB160 was initiated in Brazil in late 2018 (ClinicalTrials.gov Identifier: NCT03330899).

## Supporting information

S1 Data Set HAI titers raw data and statistical analysis. (XLSX)

S1 Checklist NC3Rs ARRIVE guidelines 2014. (PDF)
